# Berberine affects mitochondrial activity and cell growth of leukemic cells from chronic lymphocytic leukemia patients

**DOI:** 10.1038/s41598-020-73594-z

**Published:** 2020-10-05

**Authors:** Silvia Ravera, Fabio Ghiotto, Claudya Tenca, Elena Gugiatti, Sara Santamaria, Bernardetta Ledda, Adalberto Ibatici, Giovanna Cutrona, Andrea N. Mazzarello, Davide Bagnara, Martina Cardillo, Daniela Zarcone, Zbigniew Darzynkiewicz, Ermanno Ciccone, Franco Fais, Silvia Bruno

**Affiliations:** 1 Department of Experimental Medicine, University of Genoa, 16132 Genoa, Italy; 2 Department of Health Sciences, University of Genoa, 16132 Genoa, Italy; 3 Hematology Unit and Bone Marrow Transplantation, IRCCS Ospedale Policlinico San Martino, 16132 Genoa, Italy; 4 Experimental Immunology, The Feinstein Institute for Medical Research, North Shore-Long Island, Manhasset, NY USA; 5 Department of Pathology, Brander Cancer Research Institute, New York Medical College, Valhalla, NY USA; 6 Molecular Pathology Unit, IRCCS Ospedale Policlinico San Martino, 16132 Genoa, Italy

**Keywords:** Cancer, Cell biology, Oncology

## Abstract

B-cell chronic lymphocytic leukemia (CLL) results from accumulation of leukemic cells that are subject to iterative re-activation cycles and clonal expansion in lymphoid tissues. The effects of the well-tolerated alkaloid Berberine (BRB), used for treating metabolic disorders, were studied on ex-vivo leukemic cells activated in vitro by microenvironment stimuli. BRB decreased expression of survival/proliferation-associated molecules (e.g. Mcl-1/Bcl-xL) and inhibited stimulation-induced cell cycle entry, irrespective of *TP53* alterations or chromosomal abnormalities. CLL cells rely on oxidative phosphorylation for their bioenergetics, particularly during the activation process. In this context, BRB triggered mitochondrial dysfunction and aberrant cellular energetic metabolism. Decreased ATP production and NADH recycling, associated with mitochondrial uncoupling, were not compensated by increased lactic fermentation. Antioxidant defenses were affected and could not correct the altered intracellular redox homeostasis. The data thus indicated that the cytotoxic/cytostatic action of BRB at 10–30 μM might be mediated, at least in part, by BRB-induced impairment of oxidative phosphorylation and the associated increment of oxidative damage, with consequent inhibition of cell activation and eventual cell death. Bioenergetics and cell survival were instead unaffected in normal B lymphocytes at the same BRB concentrations. Interestingly, BRB lowered the apoptotic threshold of ABT-199/Venetoclax, a promising BH3-mimetic whose cytotoxic activity is counteracted by high Mcl-1/Bcl-xL expression and increased mitochondrial oxidative phosphorylation. Our results indicate that, while CLL cells are in the process of building their survival and cycling armamentarium, the presence of BRB affects this process.

## Introduction

During their migration between peripheral blood and lymphoid tissues, CLL cells undergo iterative rounds of converting to quiescence while in the periphery and re-activation with subsequent clonal expansion while in lymphoid proliferation centers mostly within secondary lymphoid tissues, where multiple molecular interactions with antigen and microenvironment contribute to leukemic B cell survival and proliferation. Drugs that are both cytotoxic to resting CLL cells and that are also able to inhibit CLLs’ activation and subsequent proliferation in lymphoid microenvironment would be beneficial for the treatment of this still incurable disease.

CLL cells strongly rely for survival and proliferation on mitochondrial activity. Indeed, unlike normal B cells, CLL cells store lipids and generate energy by utilizing fatty acids in addition to glucose^1,2^. Unlike other cancers, they do not appear to follow the Warburg effect, since they do not activate effective compensatory lactate production^3^. These observations corroborate the notion that CLL cells strongly depend on mitochondrial oxidative phosphorylation (OxPhos) for their bioenergetics^4,5^. In particular, OxPhos and mitochondrial functions are crucial for leukemic cell protection by the microenvironment and maintenance of intracellular redox homeostasis^6^, and were proposed as potential targets for therapeutic interventions in CLL.

Berberine (BRB), an alkaloid with anti-hyperglycemic and hypolipidemic properties, was recently shown to inhibit cellular lipogenesis, and respiratory complex I activity, exerting antiproliferative activity against tumor cell lines and tumor xenotransplants^7–10^, through mechanisms involving mitochondrial functions^11,12^. We, therefore, explored the in vitro cytotoxic and cytostatic effects of BRB on circulating leukemic cells derived *ex-vivo* from the peripheral blood of CLL patients and cultured in the presence of activating microenvironment stimuli.

## Results

The study was conducted on quiescent leukemic cells and on cells stimulated in vitro by lymphoid tissue-mimicking microenvironment stimuli (CD40L + IL-4 and CpG-ODN2006 + IL-15)^13,14^. CLL samples were derived from patients with heterogeneous clinical and molecular prognostic markers, including patients with aggressive disease (Binet B and C) or with unfavorable prognostic markers (i.e. unmutated IGHV, high CD38 levels, 17p deletion and TP53 or SF3B1 mutations) (Supplementary Table S1).

**Figure 1:**
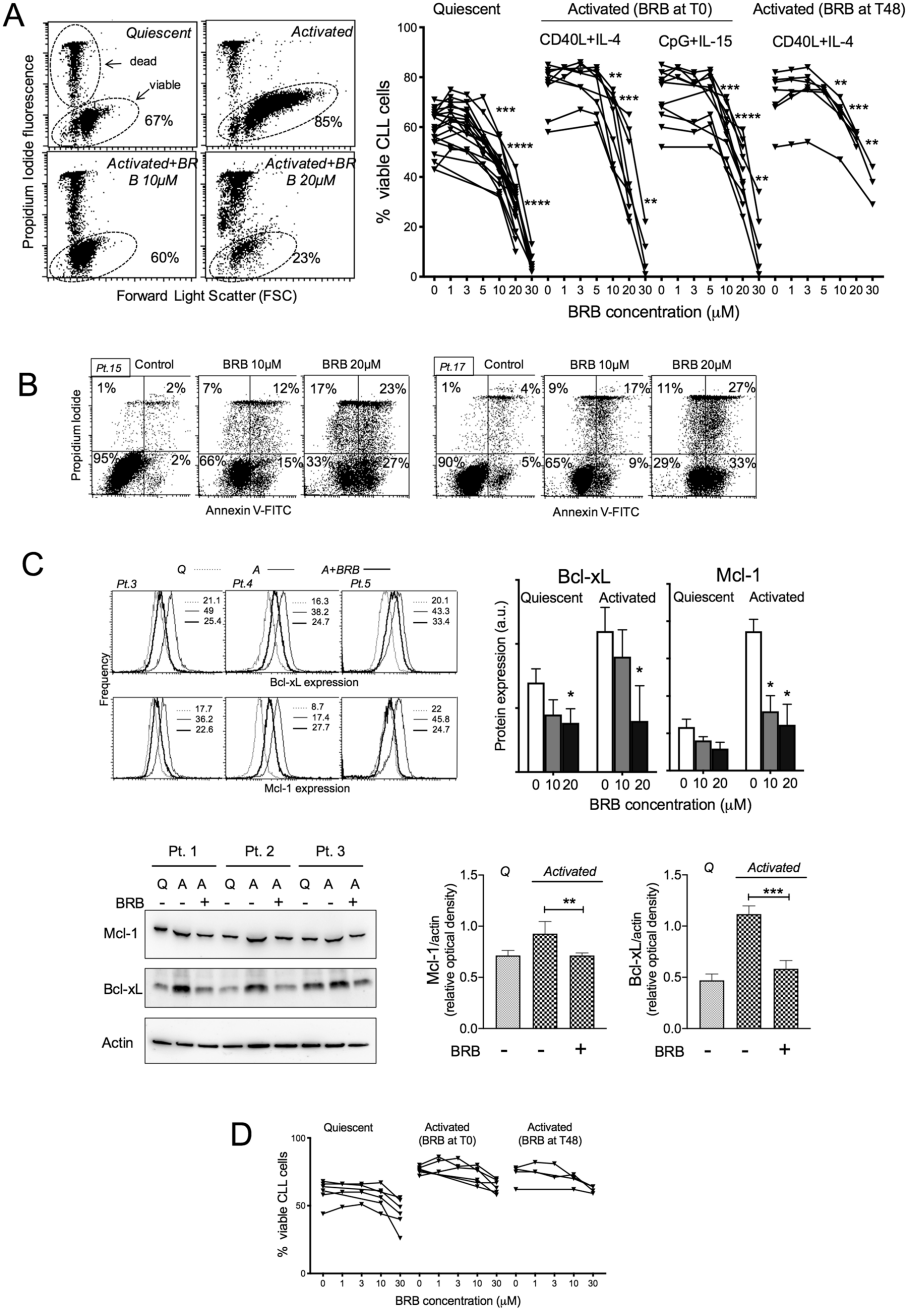
BRB affects CLL cell viability. (**A**) Left: Flow cytometric dot plots of Propidium Iodide (PI) fluorescence versus Forward Light Scatter (FSC) for the determination of live (intact plasma membrane, PI negative) and dead (disrupted plasma membrane, PI positive) cells, from one CLL patient harboring 17p13 deletion. The cells were either quiescent or stimulated by CpG/ODN2006 + IL-15 and treated with BRB 10 and 20 μM for 48 h. Drug treatment of activated cells started simultaneously with stimulation. Note that the increase of FSC (i.e. cell size) after stimulation was inhibited by the presence of BRB. Right: Cell viability by the PI exclusion test is summarized for leukemic samples from 20 CLL patients, either untreated or treated with BRB at the indicated concentrations, for 48 h. Cell activation was achieved either with CD40L-NIH-3T3 + IL-4 or CpG/ODN2006 + IL-15, as indicated. BRB treatment started at stimulation time (T0), or 48 h after stimulation (T48h). Statistical significance of differences evaluated by a two- sided Wilcoxon signed rank test. **P ≤ 0.01; ***P ≤ 0.001; ****P ≤ 0.0001. (**B**) Left: flow cytometric AnnexinV-FITC/PI dot plots of leukemic cells from two CLL patients, harboring 17p13 deletion (left) and TP53 mutation (right), stimulated with CpG + IL-15, treated simultaneously with BRB and analyzed 48 h later. (**C**) Upper left: flow cytometric frequency histograms of Mcl-1 and Bcl-xL expression in leukemic cells of three CLL patient, two of which harbored 17p13 deletion and other chromosomal abnormalities. Cells were unstimulated (Q, quiescent, dotted line) or stimulated (A, activated), in the absence (thin continuous line) or presence of 20 μM BRB (thick continuous line). The drug was added simultaneously to stimulation (CD40L-NIH-3T3 + IL-4) and cells collected 48 h later. Histograms contain only cells within the ‘live gate’, namely the flow cytometric ‘high-FSC/low SSC’ gate, that contains only cells with intact ∆Ψ, intact plasma membrane and no caspase 3 activation (see Materials and Methods and^13^). Upper right: summary of data from samples of six CLL patients, both quiescent and activated. Statistical significance of differences evaluated by a two- sided Wilcoxon signed rank test. *P ≤ 0.05. Lower left: Expression of Bcl-xL and Mcl-1 by WB analysis, in leukemic cells from three CLL patients, in unstimulated (Q, quiescent) or stimulated (A, activated) cells in the absence or presence of BRB. The drug was added simultaneously to CpG + IL-15, and cells collected 18 h later. Lower right: Optical density of protein levels was quantitated by densitometric analysis and is reported relative to actin levels. Statistical significance of differences evaluated by one-way ANOVA followed by Bonferroni post hoc test. **P ≤ 0.01; *** *P* ≤ 0.001. (**D**) Cell viability on quiescent and stimulated normal B lymphocytes, purified from peripheral blood of 6 healthy donors, and treated with BRB for 48 h. BRB treatment started at the time of stimulation (T0), or 48 h after stimulation (T48h). No significant differences were observed.

We observed a significant cytotoxic activity at concentrations of BRB ≥ 10 μM both on quiescent and stimulated CLL cultures (Fig. 1A), which was associated with apoptosis as indicated by annexinV measurements (Fig. 1B). The drug was more cytotoxic when added at the beginning of activation (T0) than when it was administered to cells in overt proliferation (T48h). Since leukemic cell activation and cell cycle entry are crucial for CLL disease progression, we were particularly interested in the effects of BRB on the early stages of cell activation. In these samples, the presence of BRB affected the expected up-regulation of anti-apoptotic Bcl-2 family members Mcl-1 and Bcl-xL (Fig. 1C), known to be particularly relevant for chemoresistance in CLL cells^15–17^. Also, BRB affected the stimulation-induced up-regulation of adhesion proteins and homing molecules (Supplementary Fig. 1S), known to activate Mcl-1 and Bcl-xL expression and to promote CLL disease development^18–21^.

It is important to highlight that the analysis of protein expression by flow cytometry was restricted to viable leukemic cells only, gating out apoptotic cells by light scattering signals or propidium iodide uptake (see Materials and Methods). These CLL cells that have still intact plasma membrane and do not express activated caspase 3^13^.

**Figure 2:**
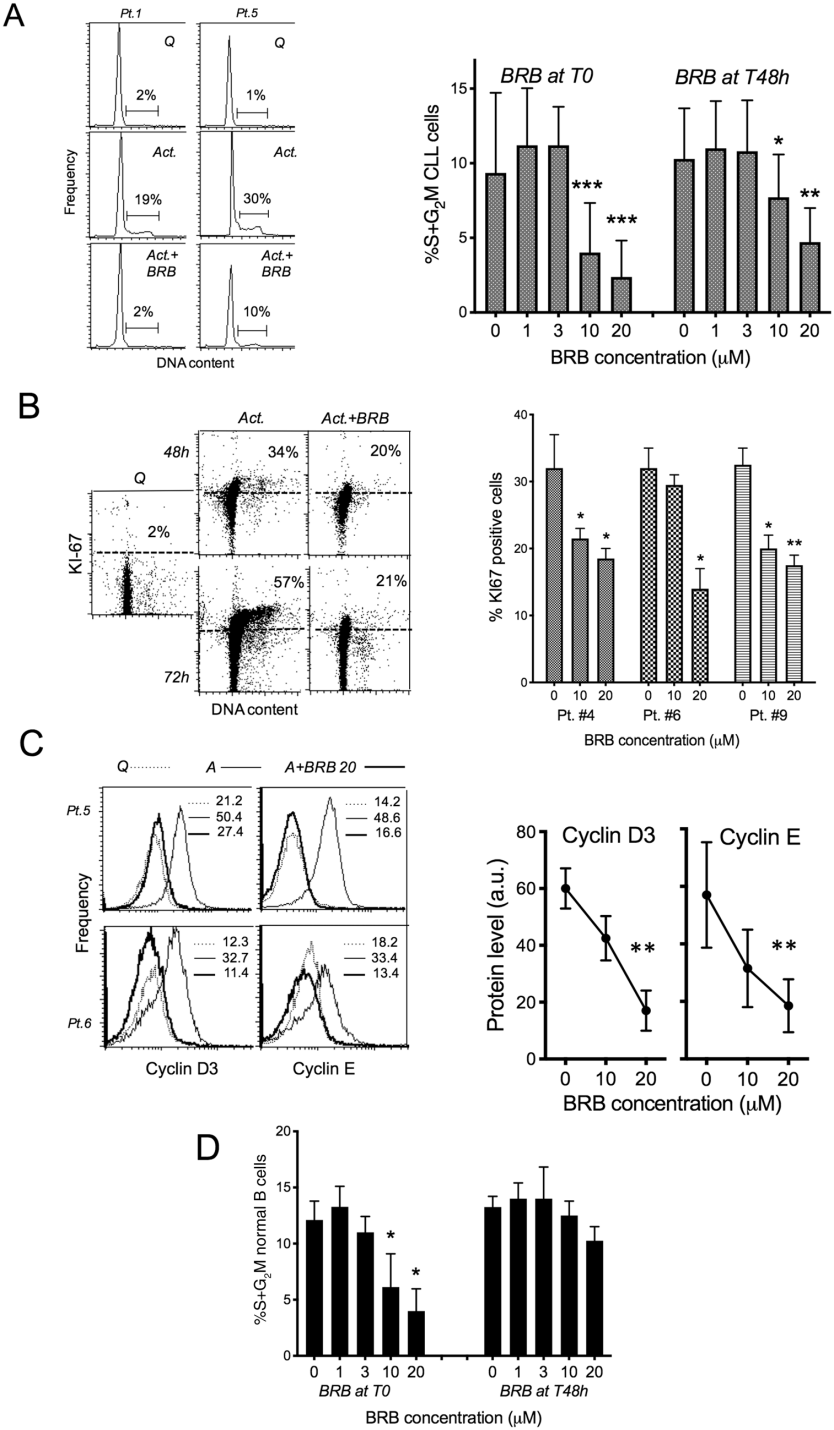
BRB affects CLL cell activation and cell cycle entry. (**A**) Left: flow cytometric DNA content histograms of leukemic cells from one CLL patient with mutated TP53. The cells were either quiescent or stimulated by CpG/ODN2006 + IL-15 and treated with BRB 20 μM for 48 h. Drug treatment of activated cells started simultaneously with stimulation. Right: Percentage of CLL cells in the S + G2M phase of the cell cycle, evaluated from flow cytometric DNA content histograms of samples from 17 CLL patients, stimulated and treated with BRB for 48 h. BRB treatment started at the time of stimulation (T0), or 48 h after stimulation (T48h). Statistical significance of differences by non-parametric t-test. *P ≤ 0.05; **P ≤ 0.01; ***P ≤ 0.001. (**B**) Left: flow cytometric DNA/KI67 bivariate plots of leukemic cells from one CLL patient with mutated SF3B1, treated with 20 μM BRB at the time of CpG + IL-15 stimulus and analyzed after 48 and 72 h. Right: Percentage of Ki-67 positive cells in leukemic cell cultures of three CLL patients, evaluated from flow cytometric DNA/KI67 bivariate plots of samples stimulated and treated simultaneously with BRB for 48 h. Statistical significance of differences by non-parametric t-test. *P ≤ 0.05; **P ≤ 0.01. (**C**) Left: frequency histograms of cyclin D3 and cyclin E expression in leukemic cells from two CLL patients, either quiescent or stimulated by CpG/ODN2006 + IL-15 and treated with BRB 20 μM for 48 h. Right: Percentage of cyclin D3 and cyclin E positive cells in CLL cultures stimulated and treated simultaneously with BRB for 48 h. Cells were processed for intracellular immunofluorescence and analyzed by flow cytometry within the ‘live’ gate. Statistical significance of differences by non-parametric t-test. **P ≤ 0.01. (**D**) Percentage of normal B lymphocytes residing in the S + G2M phase of the cell cycle, evaluated from flow cytometric DNA content histograms of samples from 6 healthy donors. Cells were stimulated and treated for 48 h with BRB either at beginning of stimulation (T0) or 48 h after stimulation (T48). Statistical significance of differences by non-parametric t-test. *P ≤ 0.05.

BRB affected cell cycle entry and proliferation, as demonstrated by observation on cell cycle associated parameters that increase their levels after microenvironment stimuli. The rise of the percentage of S + G2M phase cells 48 h after stimulation was inhibited by BRB (Fig. 2A). The reduced rise of expression of Ki-67, the proliferation marker which is maximally expressed during late-S phase and in G2, is also consistent with an anti-proliferative effect of BRB (Fig. 2B). D3 and E type cyclins have a key role in CLL for the induction of G1 progression and G1/S transition^22,23^. Their activation-induced increase was impaired by BRB (Fig. 2C).

Normal B lymphocytes were less sensitive to the alkaloid cytotoxic activity (Fig. 1D). Cell viability was totally unaffected at the doses that induced apoptosis in CLL cells. Cell proliferation of normal B cells was partly inhibited when BRB was present during the initial activation stages, while it was virtually unaffected when BRB was administered later on, i.e. on cells already in overt proliferation (Fig. 2D). This observation suggests that BRB might affect normal lymphocyte preferentially during the early stages of mitogenic stimulation while clearly proliferating cells remain resistant to its cytostatic effect.

Since CLL cells rely on mitochondrial OxPhos for their bioenergetics, we envisaged that the inhibitory effects of BRB on viability and cell proliferation might be mediated by induction of mitochondrial dysfunction and aberrant cellular energetic metabolism.

**Figure 3:**
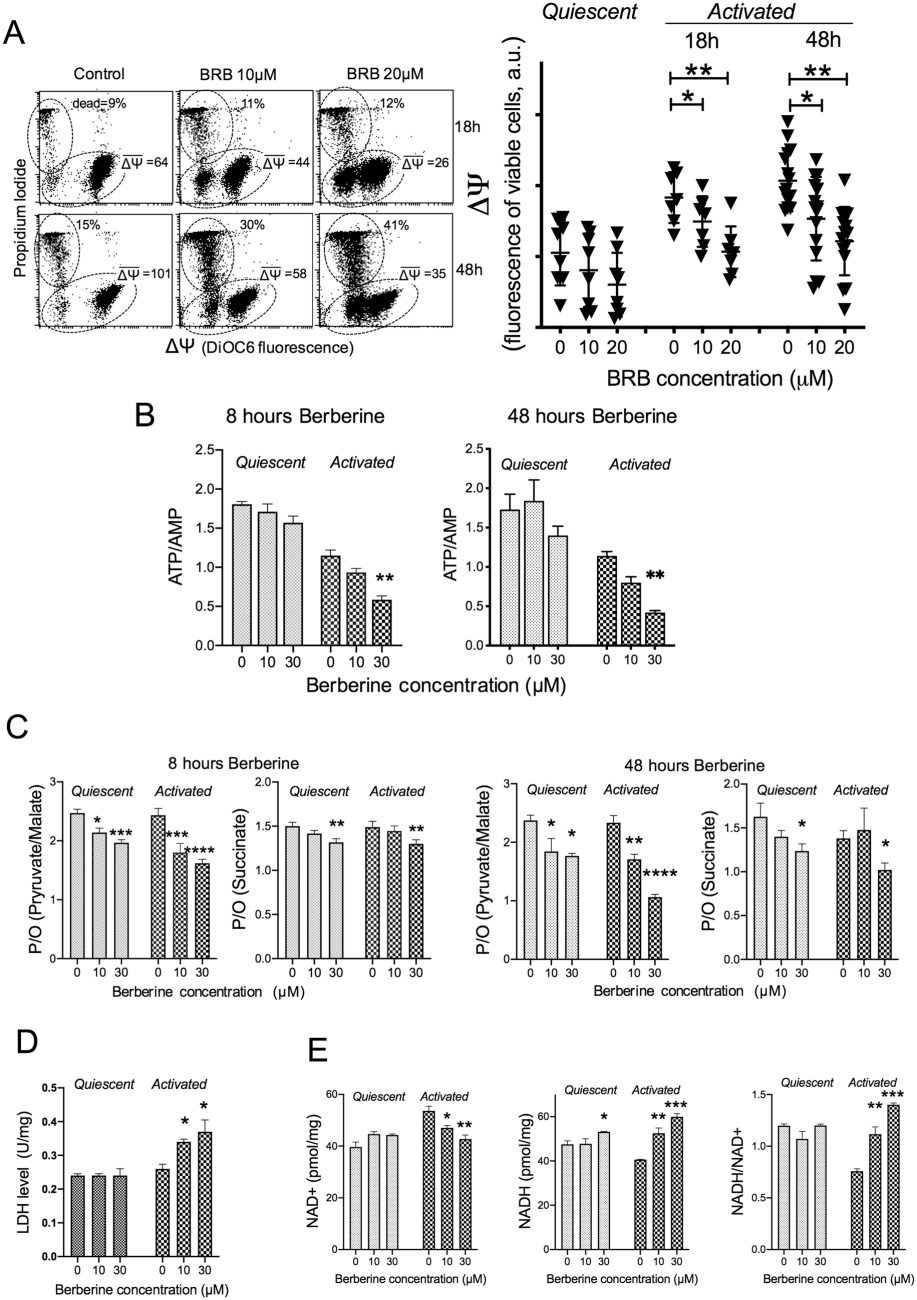
BRB affects CLL cell energetic metabolism. (**A**) Left: Mitochondrial transmembrane potential ΔΨ evaluated by fluorescence of 3,3′-dihexyloxacarbocyanine iodide (DiOC_6_) of CLL cells counterstained with Propidium Iodide to identify live/dead cells. The flow cytometric dot plots of DiOC6/PI fluorescence are displayed for one CLL sample stimulated in the absence or presence of 10 μM or 20 μM BRB and measured after 18 and 48 h. The following subpopulations: can be identified DiOC6^pos^/PI^neg^ cells (intact ∆Ψ), DiOC6^neg^/PI^neg^ cells (dissipated ∆Ψ but still intact plasma membrane) and DiOC6^neg^/PI^pos^ cells (dead cells). We report the % of dead cells and the geometric mean of DiOC_6_ for the PI^neg^ subpopulation (both DiOC6^pos^ + DiOC6^neg^, i.e. ΔΨ of the viable cells). Right: mean ΔΨ of viable cells in unstimulated CLL samples (quiescent) treated with BRB for 48 h, and stimulated samples treated for 18 h (n = 7) and 48 h (n = 15). Statistical significance of differences by non-parametric t-test. *P ≤ 0.05; **P ≤ 0.01. (**B**) Energy status evaluated as the ATP/AMP ratio of quiescent (n = 4) and stimulated (n = 4) CLL samples in response to 8 h or 48 h BRB treatment. (**C**) OxPhos coupling evaluated as the ratio between ATP synthesis and oxygen consumption (P/O ratio) on quiescent (n = 4) and stimulated (n = 4) CLL samples in response to 8 h or 48 h BRB treatment. Pyruvate + malate or succinate were used to activate the pathways triggered by Complex I or Complex II, respectively. (**D**) Lactate dehydrogenase activity (LDH) in quiescent (n = 4) and stimulated (n = 4) CLL samples in response to 48 h BRB treatment. (**E**) NAD^+^ and NADH concentrations, and the consequent NADH/NAD^+^ ratio, estimated in quiescent (n = 4) and stimulated (n = 4) CLL samples in response to 48 h BRB treatment. For Panels (**B**–**E**), statistical significance of differences evaluated by one-way ANOVA followed by Bonferroni post hoc test. *P ≤ 0.05; **P ≤ 0.01; *** *P* ≤ 0.001;****P ≤ 0.0001.

We observed that BRB inhibited the stimulation-induced increase of mitochondrial transmembrane potential (ΔΨ) (Fig. 3A), and induced ATP depletion with consequent AMP accumulation, occurring already 8 h after BRB addition (Fig. 3B). These observations are consistent with the notion that BRB exerts an inhibitory action on CLL via mitochondria likely through their respiratory chain activity, as previously demonstrated in other cellular models^12^. In support of the above, by analyzing the effects of BRB on mitochondrial respiration, we found that BRB produced a dose-dependent inhibition of oxygen consumption in activated CLL cells, with both complex I–linked (pyruvate/malate) and complex II–linked substrates (succinate) (Supplementary Figure S2). The activity of F_o_-F_1_ ATP synthase, the enzyme that uses the proton gradient across mitochondrial membrane to produce ATP, was also affected by BRB (Supplementary Figure S2). However, the level of inhibition of ATP synthase activity was higher than the inhibition of oxygen consumption, as proven by the decreased P/O ratio (Fig. 3C) This indicated a mitochondrial uncoupling status occurring well before cellular dysfunction and eventual apoptosis. Thus, it can be inferred that oxygen consumption inhibition and ATP depletion provoked by BRB at concentrations ≥ 10 μM might have a role in the impairment of cell cycle entry and proliferation.

Alterations of the aerobic metabolism normally redirect cells to an increase of lactic fermentation, to restore at least in part ATP production and recycle the NADH produced during glycolysis. Indeed, we observed that the activity of lactate dehydrogenase (LDH) was higher in activated BRB-treated cells (Fig. 3D). However, the levels of the NADH/NAD^+^ ratio were also increased (Fig. 3E), indicating that the BRB-induced anaerobic glycolytic flux was not sufficient to convert all the coenzyme reduced during the glycolytic process. Indeed, NADH levels in BRB cells remained higher than in control ones, while regeneration of NAD^+^ levels, necessary for proper glycolytic flux, was impaired (Fig. 3E). In other words, the tumor cells attempt to compensate the BRB-induced defect of aerobic metabolism by enhancing the anaerobic glycolysis, but the process of NAD^+^ regeneration seems to be not efficient enough. Conversely to CLL, energetic and glucose metabolism features were unaffected in normal B lymphocytes by these concentrations of BRB (Supplementary Figure S3).

**Figure 4:**
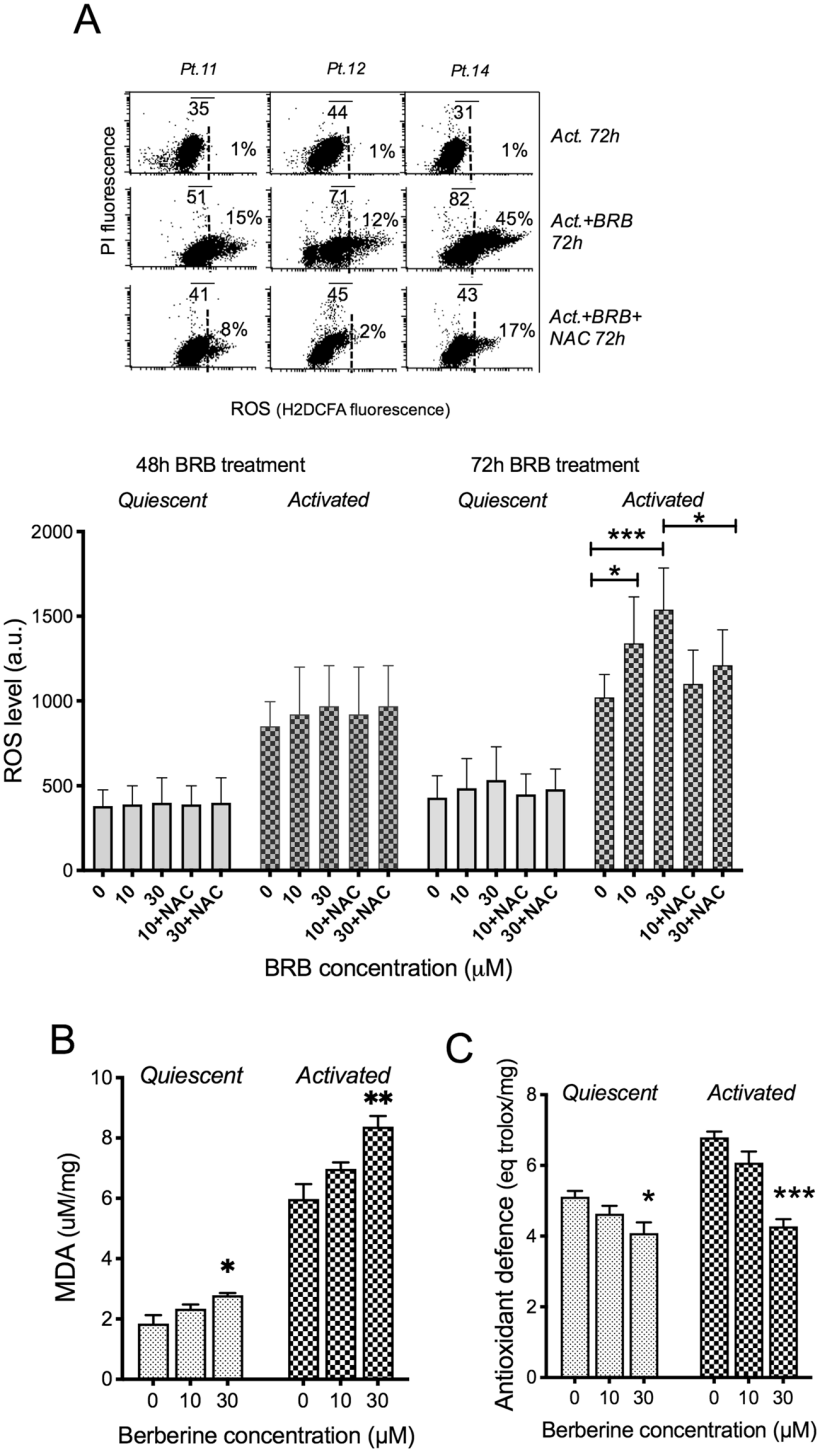
BRB affects CLL cell redox homeostasis. (**A**) Left: flow cytometric frequency histograms of H2DCFDA fluorescence in leukemic cells from one CLL patient, stimulated, treated with BRB and analyzed after 48 and 72 h (protective NAC was added as internal control). Right: ROS levels of n = 6 quiescent and stimulated CLL samples in response to 48 h and 72 h BRB treatment, as assessed by flow cytometric fluorescence of H2DCFDA stained cells, analyzed after gating out dead cells. Statistical significance of differences by non-parametric t-test. *P ≤ 0.05; **P ≤ 0.01; ***P ≤ 0.001. (**B**) Malondialdehyde (MDA) level, evaluated as a marker of lipid peroxidation, in quiescent (n = 4) and stimulated (n = 4) CLL samples in response to 48 h BRB treatment, by the thiobarbituric acid reactive substances (TBARS) method^36^. Statistical significance of differences by one-way ANOVA followed by Bonferroni post hoc test. *P ≤ 0.05; **P ≤ 0.01. (**C**) Antioxidant defense evaluated in quiescent (n = 4) and stimulated (n = 4) CLL samples in response to 48 h BRB treatment, by measuring the Total Antioxidant Capacity (TAC). The concentration of small molecule and protein antioxidants is expressed in Trolox equivalents, using the vitamin E analog Trolox as an antioxidant standard. Statistical significance of differences by one-way ANOVA followed by Bonferroni post hoc test. *P ≤ 0.05; ***P≤ 0.001.

Uncoupled OxPhos promotes the generation of ROS. Twenty-four hours after the full signs of mitochondrial dysfunction elicited by BRB, we observed increased levels of reactive oxygen intermediates in stimulated CLL cells treated with BRB (Fig. 3A). CLL cell cultures previously conditioned with antioxidants were less sensitive to BRB for ROS generation and cell cycle entry (Fig. 4A). This oxidative stress, further corroborated by the observed increase of lipid peroxidation (Fig. 4B), was not counterbalanced by antioxidant defense mechanisms that appeared to be affected themselves by BRB treatment (Fig. 4C). We may envisage that the cytotoxic action of BRB at 10–30 μM might be mediated, at least in part, by BRB-induced surplus production of mitochondrial superoxide leading to oxidative damage, and eventually to cell death, as previously observed in tumor cell lines^7,24^. ROS levels in normal B lymphocytes, instead, remained unaltered after BRB treatment (Supplementary S4).

**Figure 5:**
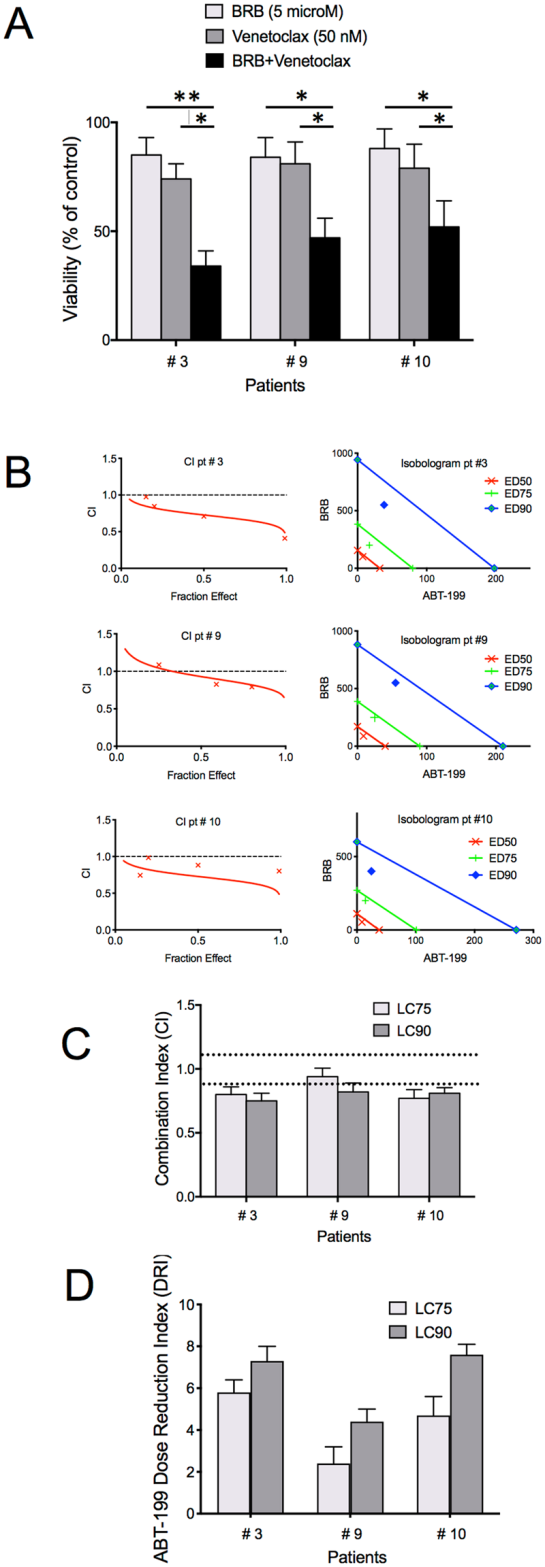
BRB potentiates the cytotoxic activity of Venetoclax on CLL cells. (**A**) BRB and/or ABT-199 cytotoxicity (42 h) on activated CLL cultures of three CLL patients. Cells displayed increased sensitivity to drug combinations, if compared to either drug alone, as demonstrated by the statistically significant difference evaluated by a two- sided Wilcoxon signed rank test. *P ≤ 0.05;** *P* ≤ 0.01. (**B**) Combination index (CI) curves and isobolograms computed by the Chou–Talalay model (CalcuSyn software, Biosoft, Cambridge) from dose–effect profiles of activated leukemic cells treated for 42 h with increasing concentrations of BRB (1–10 μM), ABT-199 (10–100 nM) or BRB/ABT-199 at constant ratios. CI measures drug interaction effects: additive: 0.9 ≤ CI ≤ 1.1, synergism: CI < 0.9 and antagonism: CI > 1.1. Isobolograms: the x- and y-axes represent the doses of ABT-199 and BRB, respectively. The intercepts of the three lines on x- and y-axes represent the dose of the same efficacy when the two drugs are used alone, which are here expressed as half, 75% and 90% effective dose (i.e. ED50, ED75 and ED90). Additive: point on the line, synergism: point below the line, antagonism: point above the line. (**C**) CI values at the ‘fractional effect levels’ LC75 and LC90 (concentration lethal to 75% and 90% of CLL cells, respectively). Dotted lines indicate CI = 0.9 and CI = 1.1. (**D**) Dose Reduction Index (DRI) values at LC_75_ and LC_90_ computed for samples of three CLL patients.

The anti-Bcl-2 drug ABT-199/Venetoclax is a promising anti-CLL agent that demonstrated potent clinical activity also in CLL cases with poor prognosis^25^. However, its cytotoxic activity is impaired when Mcl-1 and Bcl-xL are overexpressed^26^. Also, therapeutic resistance is recently emerging^27^. This resistance was mainly ascribed to the evolution of leukemic clones that display increased Mcl-1 expression and reprogrammed cellular energy metabolism, with particular regard to enhanced OxPhos activity^28^. We thus assayed combination treatments with BRB and ABT-199, given that our in vitro data indicate that BRB depressed leukemic cells’ activation and cell cycle entry possibly via altered mitochondrial activity and down-regulation of Mcl-1/Bcl-xL expression. Significant potentiation of ABT-199 cytotoxicity by BRB was observed in activated CLL cells (Fig. 5A). To elucidate whether BRB/ABT-199 drug combination was synergic or just additive, we performed cytotoxicity experiments at multiple drug concentrations and applied the Chou-Talalay model. Combination Index (CI) and isobolograms demonstrate a ‘synergic’, though not striking, effect for BRB/ABT-199 drug combination in most cases (Fig. 5B-C).

Based on the dose-effect curves of drugs alone and in combination it is possible to also calculate a Dose Reduction Index (DRI), namely a measure of how much the dose of each drug in a synergistic combination may be reduced for a given effect level compared with the doses of each drug alone. We found that BRB sensitized the activated CLL cells to the cytotoxic action of Venetoclax and significantly decreased the efficacious dose of Venetoclax by a factor ranging from 2 to 7 (Fig. 5D).

## Discussion

In this study we addressed the in vitro effects of BRB on CLL cells, quiescent or stimulated by microenvironment stimuli to become activated blasts that enter the cell cycle. We found that BRB was cytotoxic to quiescent CLL cells and, most strikingly, that the drug inhibited leukemic cell activation induced by microenvironment stimuli. These cytostatic effects of BRB on leukemic cells subjected to activation stimuli were demonstrated by inhibition of cell cycle entry and rise of proliferation-associated molecule expression. The stimulation-induced up-regulation of adhesion/homing molecule expression was also impaired by BRB. These molecules have a crucial role in eliciting mitotic activity and cell homing when leukemic cells reside in lymphoid tissues and play a pivotal role in the development and progression of CLL. In particular, CD44 promotes disease progression and apoptosis resistance through Mcl-1 up-regulation^18^. Adhesion molecules CD54 (ICAM-1) and CD58 (LFA-3) are associated with CLL disease progression^19^. CXCR4(CD184) and CD62L promote CLL cell survival and activation of leukemic cells when they are in lymph node and bone marrow^20,21^.

These responses to BRB were recorded before cell death, since the flow cytometric analysis was restricted to viable cells only, indicating that BRB down-regulated the expression of these proteins before mitochondrial perturbation and cell death.

Sensitivity of CLL sample to BRB was not found to correlate with clinical parameters (Binet disease stage) nor with molecular prognostic markers (mutational status of the IGHV, CD38 expression). Whether this lack of correlation indicates that BRB sensitivity is invariant with respect to disease aggressiveness, or if it is simply due to a small sample size, will be disclosed by further study, which will expand the data and increases statistical robustness. Nevertheless, it has to be remarked that BRB affected viability and proliferation also of CLL cells from patients with markers of aggressive disease, such as those harboring 17p deletion, TP53 and SF3B1 mutations, or other chromosomal abnormalities known to confer chemoresistance.

The cytotoxic and cytostatic effects of BRB might be caused, at least in part, by the observed mitochondrial dysfunction and the associated oxidative stress production. It is not known whether mitochondrial structures are directly or indirectly affected by the drug. Nevertheless, it is notable that, in solid tumors, BRB administered at the μM-range concentrations was documented to accumulate selectively in mitochondria^29^.

The BRB-induced defect of the mitochondrial aerobic metabolism was not compensated by enhanced anaerobic glycolysis and NAD^+^ regeneration. It produced instead increased levels of mitochondrial superoxide, as reported in other cell types as well^7,24^. Peripheral blood lymphocytes from healthy donors, either quiescent or activated, were instead insensitive to the cytotoxic action of BRB at the doses that were effective on CLL cells. This leukemia-selective action of the drug could depend on the equilibrium between its ROS generation effects and the cellular antioxidant ability to counterbalance the oxidative stress. Our data show that the basal levels of ROS and antioxidant defenses were lower in normal B lymphocytes than in CLL cells (Fig. 4 and S4), suggesting that mitochondrial metabolism is more efficient in normal than in leukemic cells. When treated with BRB, CLL cells displayed a lower antioxidant response in comparison to normal B cells. This could have induced the activation of a vicious cycle in which ROS determined a damage on the inner mitochondrial membranes, exacerbating the production of oxidative stress and the impairment of the OxPhos. By contrast, in normal B cells, the BRB-induced increment of ROS production was balanced by an adequate antioxidant defense, avoiding the mitochondrial damage and the alteration of energy metabolism. further suggesting that mitochondrial metabolism is more efficient in normal than in leukemic cells.

It has also to be recalled that leukemic cells rely on OxPhos for their bioenergetics needs, more than normal cells do^2,4^. Therefore, damage to the mitochondrial activity might lead to more dreadful consequences in CLL cells. Interestingly, CLL cells are also more addicted, for their survival and proliferation, to the protective activity of Bcl-2 anti-apoptotic proteins compared to normal cells^30^. Oncogene activation, cell cycle checkpoint violation, genomic instability are insults that normally provoke death signals. Cancer cells counteract them by using apoptotic block mechanisms, one of which is the increased activity of anti-apoptotic proteins. The anti-apoptotic proteins of the Bcl-2 family sequester and hold in check large amounts of pro-apoptotic BH3-only proteins in CLL cells^30^. Instead, the survival of nonmalignant cells does not normally depend on anti-apoptotic proteins^31^ . Thus, CLL cells are more addicted to the protective activity of Bcl-2 anti-apoptotic members compared to normal cells, and are therefore more sensitive to agents that lower anti-apoptotic protein expression and trigger the consequent massive release of pro-death BH3-only proteins^30^. Conversely, down-regulation of the anti-apoptotic proteins is not expected to exert dramatic effects on healthy cells. Although we did not demonstrate this hypothesis in our samples, we feel that it might provide an additional model that accounts for the differential sensitivity of leukemic and normal B cells to BRB.

Though normal B lymphocytes were almost unaffected by BRB at the doses cytotoxic for CLL cells, a cytostatic effect was observed at early stages of cell cycle entry. B cells overtly proliferating were instead unaffected. This suggests that the presence of BRB might slow down normal lymphocyte activation.

BRB was able to potentiate in vitro ABT-199 cytotoxicity. This may permit the use of ABT-199 at lower doses, which clinically would decrease treatment-derived morbidity and reduce therapy-derived acquisition of clinical resistance, which appears to be driven by a progressive up-regulation of Mcl-1 and an overall increased capacity for respiration and OxPhos^28^.

BRB is a well-tolerated drug at the doses used for hypercholesterolemic and diabetic patients (1–2 g/die). At these doses the plasma concentrations of BRB and its bioactive metabolites is lower (10–50 nM) than the presently seen doses that displayed metabolic inhibition in our CLL cells^32^. However, nanotechnological strategies to improve BRB delivery to tumors are recently being successfully exploited^33^ and increased BRB cytotoxicity by 20-fold in preclinical murine models^34^. Also, drug accumulation at concentrations several fold higher (up to 30-fold) than those in blood has been demonstrated in tissues^35^. We may envisage that BRB may accumulate within lymphoid tissues, which is the site where CLL cells are being activated and where a BRB-containing milieu could hamper cell cycle entry of leukemic cells and therefore decrease the threshold of cellular sensitivity to anti-CLL drugs.

## Materials and methods

### Cells and cell cultures

CLL cells were obtained from peripheral blood of CLL patients, after informed consent according to the Declaration of Helsinki. Mononuclear cells separated by Ficoll density gradient centrifugation were assayed by flow cytometry (FACSCalibur, BD Biosciences, San Diego, CA) for standard diagnostic immunophenotyping. CLL cells were cultured in RPMI culture medium with 10% FBS at high cell density, 2–4 × 10^6^/ml. CD40-activation was achieved by a CD40L-expressing NIH-3T3 murine fibroblast cell line produced in our laboratory + IL-4 (10 ng/ml) or by CpG/ODN2006 (hTLR9 ligand) (2 microg/ml) + IL-15 (10 ng/ml). In vitro response to stimuli was confirmed flow cytometrically by expression of activation molecules, increased cell size (forward light scattering) and % cells in S + G2M cell cycle phases (DNA content histograms). It has to be mentioned that the experiments were done on CLL samples with at least 85–90% leukemic clone, and that the whole mononuclear cell population was used for the experiments. T cells, which are able to proliferate in vitro after the activation stimuli provided by activated leukemic B cells, were present during the experiments at very low proportion (rarely exceeding 10% of the whole cell population), indicating a minimal contribution to the proliferation data.

Normal B cells from healthy volunteers were purified by magnetic beads (negative-selection) and activated by co-culturing with CD40L-fibroblast + IL-4 or CpG + IL-15.

### Flow-cytometric assays for apoptosis and proliferation

Multiparameter flow-cytometric analysis of cellular viability by propidium iodide (PI) exclusion assays, expression of surface (adhesion/homing) and intracellular (KI-67, cyclins and Mcl-1/Bcl-xL) proteins, Annexin V-FITC/PI fluorescence, mitochondrial transmembrane potential (∆Ψ) and cell cycle-phase distribution by DNA content were previously described^13,14^.

The flow cytometric analysis of surface and intracellular molecule expression was always restricted to viable cells. Indeed, analysis was performed selectively on CLL cells within the flow cytometric ‘high-FSC/low SSC’ gate (called here the ‘live gate’), i.e. a gate that contains, in the case of the CLL cell systems, only cells with intact ∆Ψ, intact plasma membrane and do not express activated caspase 3^13^. When PI staining was present, the positive PI fluorescence was also used to gate out dead cells from the analysis.

### Oxygen consumption, ATP synthase activity, ATP/AMP, lactate dehydrogenase activity, NADH/NAD^+^

Oxygen consumption measures were conducted with a microelectrode (Unisense Microrespiration, Denmark) and F_o_-F_1_ ATP synthase activity with a luminometer (Glomax 20/20, Luminometer -Promega, USA), in the presence of pyruvate + malate or succinate, to activate the pathways triggered by Complex I or Complex II, respectively^36^. Intracellular ATP and AMP concentrations were evaluated spectrophotometrically following NADP reduction or NADH oxidation, while lactate dehydrogenase activity was assayed following NAD^+^ oxidation^36^. NAD^+^ and NADH concentrations, and the consequent NADH/NAD^+^ ratio, were estimated in quiescent by a colorimetric NAD/NADH assay^36^.

### Ros and antioxidants

Cellular ROS levels were assessed by flow cytometric fluorescence of H2DCFDA stained cells. Lipid peroxidation was evaluated through measurements of malondialdehyde (MDA) by the thiobarbituric acid reactive substances (TBARS) method^36^. Antioxidant defense was evaluated using the Total Antioxidant Capacity Assay Kit (cod: MAK187, Merck, Germany).

### Western blot analysis

Expression of Mcl-1 and Bcl-xL was determined by Western blot, using standard procedures. Quiescent and activated CLL cells treated or not with 20 μM BRB were lysed in the presence of a protease inhibitor cocktail for mammalian cells and total protein was measured by Bradford assay. After SDS-PAGE, performed according to the standard method on 4–20% precast gels (BioRad), proteins were transferred to a nitrocellulose membrane. The membrane was blocked for 1 h with Tris Buffered Saline (TBS) plus 0.15% Tween 20 (TBSt) containing 5% non-fat dry milk and incubated over-night at 4 °C with the following mouse monoclonal antibodies: anti-Mcl-1 (1:500, Santa-Cruz Biotechnology, sc-12756), anti-Bcl-xL (1:500, Santa-Cruz Biotechnology, sc-8392) or anti-actin (1:10,000, Thermo-Fisher MA5-11869). After washing with TBSt, the membrane was incubated with an anti-mouse IgG antibody conjugated with horse radish peroxidase (HRP) (BioRad) and developed with Clarity Western ECL Substrate (BioRad). Bands were detected and analyzed for density using an enhanced chemiluminescence system (Alliance 6.7 WL 20 M, UVITEC, Cambridge, UK) and UV1D software (UVITEC). Bands of interest were normalized for actin level in the same membrane.

### Combination cytotoxicity

Combination cytotoxicity of Berberine and ABT-199 (LC Laboratories, Woburn, MA, USA) was calculated by the Chou–Talalay method^37^(CalcuSyn software, Biosoft, Cambridge). Combination index (CI), computed from dose–effect curves of drugs alone and in combination, represents a quantitative measure of the degree of drug interaction in terms of additive effect (0.9 < CI < 1.1), synergism (CI < 0.9), or antagonism (CI > 1.1) for cellular cytotoxicity. CI value usually differs for different “fractional effect level” at which it is calculated. Here we considered two levels of cytotoxicity and calculated CI values at LC_75_ and LC_90_ (i.e. concentration lethal to 75% and 90% of CLL cells).

The Dose Reduction Index (DRI), which indicates how many times the dose of ABT-199, when used in combination with a coordinated dose of BRB (at given molar ratio), can be reduced to reach a same Fractional Effect, was calculated with the CalcuSyn software from cytotoxicity profiles of the drugs alone and in combination, according to the Chou–Talalay method^37^.

### Statistics

Non parametric statistics was applied, using the GraphPad Prism version 5.00 statistical software (GraphPad Software Inc., La Jolla, CA). Details in Figure Legends.

### Ethics approval

CLL samples were obtained from patients enrolled in the observational multicenter study (clinical trial.gov identifier NCT00917540). All experimental protocols were approved by the Comitato Etico Regionale (CER) Liguria and were performed according to the Declaration of Helsinki.

### Informed consent

Informed consent was obtained from all CLL patients.

## Supplementary information

Supplementary file1

## Data Availability

All methods were carried out in accordance with relevant guidelines and regulations. Materials, data and associated protocols are fully available upon request.

## References

[1] Rozovski U (2015). Aberrant LPL expression, driven by STAT3, mediates free fatty acid metabolism in CLL cells. Mol. Cancer Res..

[2] Adekola KU (2015). Investigating and targeting chronic lymphocytic leukemia metabolism with the human immunodeficiency virus protease inhibitor ritonavir and metformin. Leuk. Lymphoma.

[3] Martinez Marignac VL, Smith S, Toban N, Bazile M, Aloyz R (2013). Resistance to Dasatinib in primary chronic lymphocytic leukemia lymphocytes involves AMPK-mediated energetic re-programming. Oncotarget.

[4] Jitschin R (2014). Mitochondrial metabolism contributes to oxidative stress and reveals therapeutic targets in chronic lymphocytic leukemia. Blood.

[5] Vangapandu HV (2017). B-cell receptor signaling regulates metabolism in chronic lymphocytic leukemia. Mol. Cancer Res..

[6] Yosifov DY (2020). Oxidative stress as candidate therapeutic target to overcome microenvironmental protection of CLL. Leukemia.

[7] Fan XX (2018). Suppression of lipogenesis via reactive oxygen species-AMPK signaling for treating malignant and proliferative diseases. Antioxid. Redox Signal..

[8] Yan XJ (2017). Mitochondria play an important role in the cell proliferation suppressing activity of berberine. Sci. Rep..

[9] Lin YS (2019). Different mechanisms involved in the berberine-induced antiproliferation effects in triple-negative breast cancer cell lines. J. Cell. Biochem..

[10] Huang Y (2018). Berberine, a natural plant alkaloid, synergistically sensitizes human liver cancer cells to sorafenib. Oncol. Rep..

[11] Pereira CV, Machado NG, Oliveira PJ (2008). Mechanisms of berberine (natural yellow 18)-induced mitochondrial dysfunction: interaction with the adenine nucleotide translocator. Toxicol. Sci..

[12] Turner N (2008). Berberine and its more biologically available derivative, dihydroberberine, inhibit mitochondrial respiratory complex I: a mechanism for the action of berberine to activate AMP-activated protein kinase and improve insulin action. Diabetes.

[13] Bruno S (2012). N-(4-hydroxyphenyl)retinamide promotes apoptosis of resting and proliferating B-cell chronic lymphocytic leukemia cells and potentiates fludarabine and ABT-737 cytotoxicity. Leukemia.

[14] Gugiatti E (2018). A reversible carnitine palmitoyltransferase (CPT1) inhibitor offsets the proliferation of chronic lymphocytic leukemia cells. Haematologica.

[15] Veronese L (2008). Low MCL-1 mRNA expression correlates with prolonged survival in B-cell chronic lymphocytic leukemia. Leukemia.

[16] Vogler M (2009). Concurrent up-regulation of BCL-XL and BCL2A1 induces approximately 1000-fold resistance to ABT-737 in chronic lymphocytic leukemia. Blood.

[17] Pepper C (2008). Mcl-1 expression has in vitro and in vivo significance in chronic lymphocytic leukemia and is associated with other poor prognostic markers. Blood.

[18] Fedorchenko O (2013). CD44 regulates the apoptotic response and promotes disease development in chronic lymphocytic leukemia. Blood.

[19] Kimby E, Rincon J, Patarroyo M, Mellstedt H (1994). Expression of adhesion molecules CD11/CD18 (Leu-CAMs, beta 2-integrins), CD54 (ICAM-1) and CD58 (LFA-3) in B-chronic lymphocytic leukemia. Leuk. Lymphoma.

[20] Burgess M (2013). CD62L as a therapeutic target in chronic lymphocytic leukemia. Clin. Cancer Res..

[21] Burger JA, Peled A (2009). CXCR4 antagonists: targeting the microenvironment in leukemia and other cancers. Leukemia.

[22] Decker T (2002). Cell cycle progression of chronic lymphocytic leukemia cells is controlled by cyclin D2, cyclin D3, cyclin-dependent kinase (cdk) 4 and the cdk inhibitor p27. Leukemia.

[23] Sherr CJ (1996). Cancer cell cycles. Science.

[24] Hou D (2017). Berberine induces oxidative DNA damage and impairs homologous recombination repair in ovarian cancer cells to confer increased sensitivity to PARP inhibition. Cell Death Dis..

[25] Roberts AW (2016). Targeting BCL2 with venetoclax in relapsed chronic lymphocytic leukemia. N. Engl. J. Med..

[26] Choudhary GS (2015). MCL-1 and BCL-xL-dependent resistance to the BCL-2 inhibitor ABT-199 can be overcome by preventing PI3K/AKT/mTOR activation in lymphoid malignancies. Cell Death Dis..

[27] Anderson MA (2017). Clinicopathological features and outcomes of progression of CLL on the BCL2 inhibitor venetoclax. Blood.

[28] Guieze R (2019). Mitochondrial reprogramming underlies resistance to BCL-2 inhibition in lymphoid malignancies. Cancer Cell.

[29] Pereira GC (2007). Mitochondrially targeted effects of berberine [Natural Yellow 18, 5,6-dihydro-9,10-dimethoxybenzo(g)-1,3-benzodioxolo(5,6-a) quinolizinium] on K1735–M2 mouse melanoma cells: comparison with direct effects on isolated mitochondrial fractions. J. Pharmacol. Exp. Ther..

[30] Del Gaizo Moore V (2007). Chronic lymphocytic leukemia requires BCL2 to sequester prodeath BIM, explaining sensitivity to BCL2 antagonist ABT-737. J. Clin. Invest..

[31] Vo TT (2012). Relative mitochondrial priming of myeloblasts and normal HSCs determines chemotherapeutic success in AML. Cell.

[32] Spinozzi S (2014). Berberine and its metabolites: relationship between physicochemical properties and plasma levels after administration to human subjects. J. Nat. Prod..

[33] Sreeja S, Krishnan Nair CK (2018). Tumor control by hypoxia-specific chemotargeting of iron-oxide nanoparticle—Berberine complexes in a mouse model. Life Sci..

[34] Shen R, Kim JJ, Yao M, Elbayoumi TA (2016). Development and evaluation of vitamin E d-alpha-tocopheryl polyethylene glycol 1000 succinate-mixed polymeric phospholipid micelles of berberine as an anticancer nanopharmaceutical. Int. J. Nanomed..

[35] Tan XS (2013). Tissue distribution of berberine and its metabolites after oral administration in rats. PLoS ONE.

[36] Ravera S (2016). Evaluation of energy metabolism and calcium homeostasis in cells affected by Shwachman-Diamond syndrome. Sci. Rep..

[37] Chou TC, Talalay P (1984). Quantitative analysis of dose-effect relationships: the combined effects of multiple drugs or enzyme inhibitors. Adv. Enzyme Regul..

