# Supplementary material for: Berberine affects mitochondrial activity and cell growth of leukemic cells from chronic lymphocytic leukemia patients
Source: Sci Rep. 2020 Oct 5;10:16519. doi: 10.1038/s41598-020-73594-z (PMC7536443; doi:10.1038/s41598-020-73594-z)
Supplement: Supplementary file 1 — Supplementary file1 [file 41598_2020_73594_MOESM1_ESM.doc]

Supplementary File to:

**Berberine affects mitochondrial activity and cell growth of leukemic cells from chronic lymphocytic leukemia patients**

**Journal: Scientific Reports**

Silvia Ravera ^1*^, Fabio Ghiotto ^1, 6*^, Claudya Tenca ^1^, Elena Gugiatti ^1^, Sara Santamaria ^1^, Bernardetta Ledda ^2^, Adalberto Ibatici ^3^, Giovanna Cutrona ^6^, Andrea N. Mazzarello ^4^, Davide Bagnara ^1^, Martina Cardillo ^6^, Daniela Zarcone ^1^, Zbigniew Darzynkiewicz ^5^, Ermanno Ciccone ^1^, Franco Fais ^1, 6^ ^#^ and Silvia Bruno ^1#^ .

^1^ Department of Experimental Medicine, University of Genoa, Genoa 16132, Italy;

^2^ Department of Health Sciences, University of Genoa, 16132 Genoa, Italy.

^3^ Hematology Unit and Bone marrow transplantation, IRCCS Ospedale Policlinico San Martino, Genoa, 16132, Italy.

^4^ The Feinstein Institute for Medical Research, North Shore-Long Island, Experimental Immunology, Manhasset, NY, USA.

^5^  Brander Cancer Research Institute, Department of Pathology, New York Medical College, USA.

^6^ Molecular Pathology Unit, IRCCS Ospedale Policlinico San Martino, Genoa, 16132 , Italy.

Correspondence to Silvia Bruno: [silvia.bruno@unige.it](mailto:silvia.bruno@unige.it)

Figure S1: *BRB affects the up-regulation of adhesion and homing molecules induced by microenvironment stimuli.*

Upper: flow cytometric histograms of adhesion/homing molecule expression in leukemic cells from one representative CLL patients, either unstimulated (Q, quiescent, dotted line), or stimulated (A, activated) in the absence (thin continuous line) or presence of 10 μM BRB (thick continuous line). The drug was added simultaneously to stimulation (CD40L-NIH-3T3+IL-4) and cells collected 48 hours later. Histograms contain only cells within the ‘live gate’, namely the flow cytometric ‘high-FSC/low SSC’ gate, that contains only cells with intact plasma membrane and no caspase3 activation (see Materials and Methods).

Lower: Expression of adhesion and homing molecules in leukemic cells from four CLL patients, either unstimulated (Quiescent, Q) and untreated, or stimulated (Activated, A) and either untreated or treated simultaneously with 10 μM of BRB for 48 hours. For each adhesion/homing molecule, an arbitrary value of 100 was assigned to the sample that displayed the highest expression level. Data are reported as mean ± SD. Statistical significance of differences was evaluated by a two- sided Wilcoxon signed rank test. **P*≤0.05; ***P*≤0.01.

Figure S2:

*BRB affects oxygen consumption and ATP synthesis in CLL cells*

Oxygen consumption and F_o_-F_1_ ATP synthase activity of CLL samples in response to 8 hours (above, n=3 quiescent and n=3 stimulated) and stimulated 48 hours (below, n=4 quiescent and n=4 stimulated) of BRB treatment. Statistical significance of differences by one-way ANOVA followed by Bonferroni post hoc test. ***P*≤0.01; ***P≤0.001; ****P≤0.0001.

Figure S3:

*BRB does not affect normal B lymphocytes energetic metabolism.*

1. Mitochondrial transmembrane potential ΔΨ of normal B lymphocyte samples, either quiescent (n=6) and (CD40L+IL4)-stimulated (n=6), and treated for 48 hours with BRB. ΔΨ was evaluated by flow cytometric fluorescence of 3,3’-dihexyloxacarbocyanine iodide (DiOC_6_) gated on live cells. No statistical significance of differences was observed.
2. The energy status was evaluated as the ATP/AMP ratio of quiescent (n=3) and stimulated (n=3) normal B lymphocytes in response to 48 hours BRB treatment. Intracellular ATP and AMP concentrations were evaluated spectrophotometrically, with the enzyme coupling method, following the NADP reduction or NADH oxidation, respectively, at 340 nm. Statistical significance of differences by one-way ANOVA followed by Bonferroni post hoc test. No statistical significance of differences was observed.
3. Upper: The oxygen consumption of quiescent (n=3) and stimulated (n=3) normal B lymphocytes in response to 48 hours BRB treatment. The measure was conducted in the presence of pyruvate + malate or succinate, to activate the pathways triggered by Complex I or Complex II, respectively. No statistical significance of differences was observed.

Middle: The F_o_-F_1_ ATP synthase activity of quiescent (n=3) and stimulated (n=3) normal B lymphocytes in response to 48 hours BRB treatment. The measure was conducted with a luminometer in the presence of pyruvate + malate or succinate, to activate the pathways triggered by Complex I or Complex II, respectively. No statistical significance of differences was observed.

Lower: The coupling of OxPhos was evaluated as the ratio between ATP synthesis and oxygen consumption (P/O ratio) on quiescent (n=4) and stimulated (n=4) CLL samples in response to 48 hours BRB treatment, in the presence of pyruvate + malate or succinate, to activate the pathways triggered by Complex I or Complex II, respectively. No statistical significance of differences was observed.

1. Lactate dehydrogenase activity (LDH) was assayed as a marker of anaerobic glycolysis in quiescent (n=3) and stimulated (n=3) normal B lymphocytes in response to 48 hours BRB treatment, following the NAD^+^ oxidation, at 340 nm. No statistical significance of differences was observed.
2. NAD^+^ and NADH concentrations, and the consequent NADH/NAD^+^ ratio, were estimated in quiescent (n=3) and stimulated (n=3) normal B lymphocytes in response to 48 hours BRB treatmentNo statistical significance of differences was observed.

Figure S4

*BRB does not affect normal B lymphocytes redox homeostasis.*

1. ROS levels of n=6 quiescent and stimulated normal B lymphocytes in response 72 hours BRB treatment, as assessed by flow cytometric fluorescence of H2DCFDA stained cells, after gating on live cells. No statistical significance of differences was observed.
2. Malondialdehyde (MDA) level was evaluated as a marker of lipid peroxidation, in quiescent (n=3) and stimulated (n=3) normal B lymphocytes in response to 48 hours BRB treatment, by the thiobarbituric acid reactive substances (TBARS) method. No statistical significance of differences was observed.
3. Antioxidant defense were evaluated in quiescent (n=3) and stimulated (n=3) normal B lymphocytes in response to 48 hours BRB treatment, using the Total Antioxidant Capacity Assay Kit (Merck, Germany). No statistical significance of differences was observed.

Figure S5

Original blots of Figure 1 (of the main manuscript)

Table S1

CLL patient characteristics

* U-CLL : IGHV mutation ≤2%; M-CLL: IGHV mutation >2%.

** Positive if > 10% cells of leukemic clone display aberrant FISH
